# Kinase-two-hybrid: towards the conditional interactome

**DOI:** 10.15252/msb.20156107

**Published:** 2015-03-26

**Authors:** David Ochoa, Pedro Beltrao

**Affiliations:** European Molecular Biology Laboratory, European Bioinformatics Institute (EMBL-EBI)Cambridge, UK

## Abstract

The dynamics of the protein–protein interaction network and how it responds to biological perturbations remain difficult to assay by most traditional techniques. A novel kinase-dependent yeast two-hybrid framework by Stelzl and colleagues (Grossmann *et al*, 2015) provides a new prism to study how tyrosine phosphorylation regulates the changes in the interactome under varying conditions.

See also: A Grossmann et al (March 2015)

The static nodes-and-edges view of the cell's interaction network is coming to an end as different technologies are allowing us to measure how the cell changes in response to different conditions (Ideker & Krogan, 2012). While some proteins cooperate to form relatively stable molecular factories such as the polymerases or the proteasome, others join forces from time to time to respond appropriately to external or internal stimuli. Similarly to the way that signalling pathways respond to a given stimulus, the interactome perceives perturbations and mobilizes the proteins that are required for elaborating a precise response. Although our understanding of the static version of the interactome has improved considerably during the past decade (Stelzl *et al*, 2005; Gavin *et al*, 2006; Krogan *et al*, 2006), the transient interactions and the mechanisms regulating them are a lot less understood. Grossmann *et al* (2015) address this challenge by developing a method for identifying protein interactions that depend on tyrosine phosphorylation events.

Protein post-translational modifications (PTMs) such as phosphorylation are one of the many mechanisms used by the cell to exert control over protein interactions. When a residue is modified, the binding affinity of the protein increases towards certain “reader” domains that interpret the modification in the context of the whole protein (Seet *et al*, 2006). For example, 14–3–3 domains can bind to serine and threonine phosphosites, while the SH2 domains are known to recognize tyrosine phosphopeptides. These interactions can be affected by changes in cellular conditions via the activation of different protein kinases and the reversible recruitment of phospho-binding domains to phosphorylated proteins. Grossmann *et al* (2015) modified the standard yeast two-hybrid protocol (Y2H) by including active tyrosine kinases when assaying phospho-binding proteins as baits. Contrary to the canonical protocol, this system accounts for the major regulatory role of PTMs. For the first time, the authors were able to experimentally determine a large number of phospho-dependent protein interactions in a single experiment. As a result, they provided a list of 291 interactions that require tyrosine kinases and 336 interactions that are not kinase dependent.

Any new methodology requires a careful validation. Consequently, Grossmann *et al* (2015) extensively benchmarked the new yeast two-hybrid method against different validation sets. Firstly, a comparison of the identified kinase-dependent interactions with interactions previously described in the literature suggested that the majority of interactions remain to be discovered. Secondly, interactions were also mined for the presence of known tyrosine binding motifs, which explain ∽1/6 of the novel interactions. Finally, a subset of interactions was retested by co-IP assays in the presence/absence of kinases and using mutated versions of the binding domains. In summary, the findings strongly support the view that the identified interactions are dependent on phosphorylation even if the majority cannot be explained by the known specificities of the corresponding phospho-binding domains. Additional work will be required to determine why known binding linear motifs only explain a small fraction of the phosphorylation dependence of these interactions.

The authors have exemplarily analysed in more detail the interaction between TSPAN2 and the phosphotyrosine-binding proteins GRB2 and PIK3R3. They showed that mutations of tyrosine residues of TSPAN2, in particular of Y124, strongly affected the interaction to GRB2/PIK3R3. Mutation of Y124 also significantly reduced the membrane protrusion phenotype that is induced by the expression of TSPAN2 in HEK293 cells. As illustrated by these follow-up experiments, the set of phospho-dependent interactions collected by Grossmann *et al* (2015) serves as a useful resource that can be further explored by future studies.

**Figure 1:**
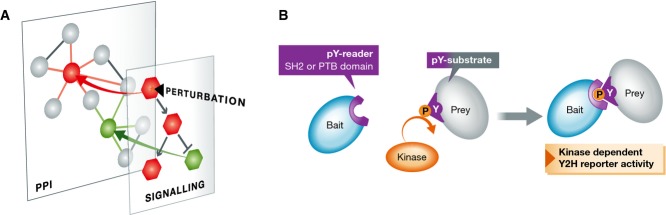
Protein interactions dependent on tyrosine phosphorylation (A) Conditional interaction networks. Signalling responses affect PTMs, such as tyrosine phosphorylation, which in turn trigger the formation of conditional (PTM-dependent) interactions. (B) Schematic of the modified Y2H assay presented by Grossman *et al* Active tyrosine kinases were added to allow detecting pY-dependent interactions between bait and prey proteins.

This work is one of the recent method developments that are extending high-throughput technologies to allow the large-scale characterization of “differential” cellular interaction networks (Ideker & Krogan, 2012). While the first decade of systems biology has charted out a detailed but static picture of the cell, these new methods will allow us to quickly determine how cells change as they react to cues. Developments in differential interaction network methods and single-cell technologies are opening up new fascinating research questions. What is the repertoire of different cellular states that exist and how specific are they? What controls the transition between states and how deterministic and stable are they? The new method developed by Grossmann *et al* (2015) will contribute to addressing these fascinating questions.
